# Atypical vascular lesions cleared with Mohs micrographic surgery

**DOI:** 10.1016/j.jdcr.2023.11.026

**Published:** 2023-12-07

**Authors:** Jose L. Cortez, Hillary Elwood, Andrew Matsumoto

**Affiliations:** a Department of Dermatology, University of New Mexico, Albuquerque, New Mexico; b Department of Dermatopathology, TriCore Reference Laboratories, Albuquerque, New Mexico

**Keywords:** angiosarcoma, atypical vascular lesions, dermatopathology, mohs micrographic surgery, radiation

## Introduction

Atypical vascular lesions (AVLs) in patients having undergone treatment for breast cancer have been described microscopically as dilated vascular spaces that often present 1-6 years after a patient undergoes radiation therapy.^1^^,^^2^ AVLs are considered benign lesions that exist on a spectrum with malignant angiosarcoma.^3^ It is unclear whether AVLs represent precursor lesions to angiosarcomas or are an independent risk factor for developing angiosarcoma.^4^ The application of Mohs surgery can be considered to achieve clear margins in select cases. Here, we describe a case of AVLs arising on a patient’s chest following radiation therapy for breast cancer cleared with Mohs micrographic surgery.

## Case report

A 33-year-old female with a history of estrogen receptor, progesterone receptor, and human epidermal growth factor receptor 2 positive left breast invasive ductal carcinoma diagnosed and treated in 2016 was evaluated by dermatology for treatment of atypical vascular lesions with Mohs surgery.

In 2017, the patient underwent neoadjuvant chemotherapy, bilateral skin sparing mastectomy with two-stage tissue expander reconstruction, and adjuvant radiation therapy. She completed her reconstruction with bilateral deep inferior epigastric artery perforator flaps that same year. In July 2022, the patient reported new skin lesions on the inferior/medial left reconstructed breast that were biopsied and found to be atypical vascular lesions. Given concern for risk of transformation to angiosarcoma, she subsequently underwent 2 excisions in September 2022 and November 2022 with surgical oncology with positive margins. The patient was presented at her hospital’s tumor board who recommended complete resection of the AVLs and close surveillance. The patient then underwent a third excision in March 2023 with plastic surgery, and margins were again positive. She was then referred to our Mohs surgery clinic for consideration of treatment with Mohs surgery.

**Fig 1:**
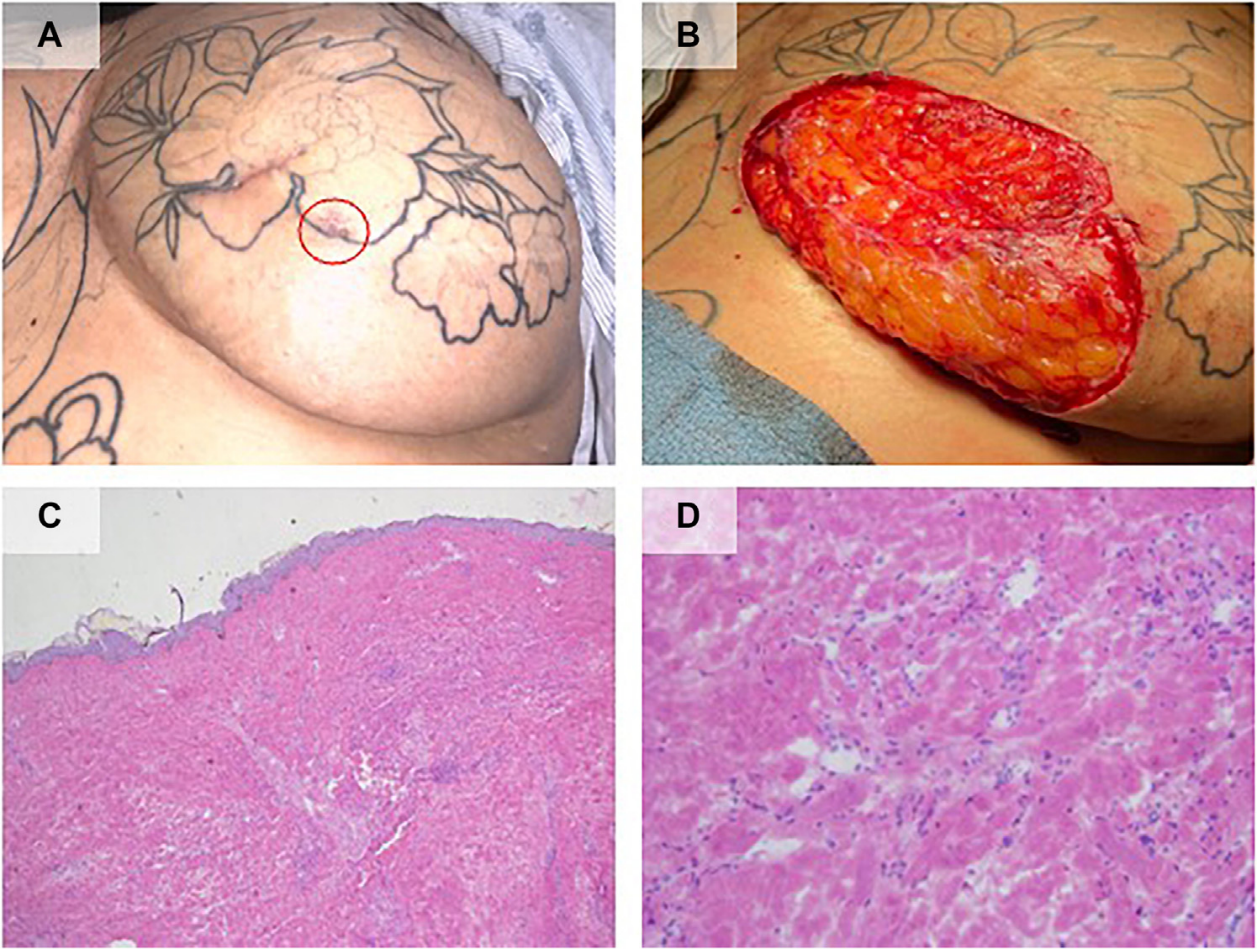
Clinical images. **A,** Left breast demonstrating well healed prior excision scars and dusky, cigarette burn, atrophic papules and plaques (*red circle*). **B,** Left breast defect measuring 8.5 × 9.5 centimeters in size with clear margins after 6 stages of Mohs micrographic surgery. Histopathology. Frozen sections stained with hematoxylin and eosin demonstrating atypical ectatic vessels in the dermis with hobnailed endothelial cells projecting into the lumina (**C**) scan and (**D**) 20× magnification.

The patient was first evaluated by dermatology in April 2023 and at the time was noted to have telangiectasias overlying the sternum/between the breasts, with multiple well healed surgical scars and purpuric plaque at the lateral and inferior edge of her most recent scar on the left breast, Fig 1, *A*. After discussion of treatment options, she underwent Mohs surgery with intraoperative histopathology demonstrating persistence of vascular ectasias/atypical vascular lesions with characteristic hobnailed endothelial cells, Fig 1, *C* and *D*. After 6 stages, a clear margin was achieved with a final defect size of 8.5 x 9.5 centimeters, Fig 1, *B*. Repair was performed by the referring plastic surgeon with full-thickness skin graft reconstruction of the defect.

## Discussion

AVLs were first described by Fineberg et al. as focal proliferations of dilated vascular spaces in 4 patients who had undergone radiation therapy for breast carcinoma.^5^ These lesions were further defined by Brenn et al and Patton et al in 2005 and 2008, respectively. Clinically, AVLs present as flesh-colored to erythematous/violaceous papules or nodules to plaques most commonly on the chest wall 1-6 years after radiation therapy.^1^^,^^2^

There are 2 histologic subtypes of AVLs described in the literature, a lymphatic and vascular type. Lymphatic AVLs are composed of predominantly thin-walled lymphatic vessels present in the superficial to mid-dermis.^2^^,^^4^ In contrast, vascular AVLs consist of superficial small capillary-sized vessels lined by hobnailed endothelial cells with extravasated red blood cells and hemosiderin, and a surrounding lymphatic component.^2^^,^^4^ Both types of AVLs are thought to arise secondary to vascular obstruction from radiation therapy and surgery, resulting in dilation of vessels.^4^ The dilated vessels present within or adjacent to the area of radiated skin and are thought to be contiguous though this has not been explicitly described in the literature.^6^

AVLs and angiosarcomas exist on a spectrum, with AVLs often being indistinguishable from well-differentiated angiosarcomas.^3^ While AVLs have a benign course, angiosarcoma has a very high mortality, so it is important to distinguish between these 2 entities. It is unclear if AVLs represent a true precursor to angiosarcoma, or whether this entity is simply an independent risk factor for the development of secondary angiosarcoma.^4^ However, there have been reports of AVLs progressing to angiosarcoma after repeated recurrences of AVLs.^7^

Given the potential for malignant transformation, it is often recommended that AVLs be treated definitively to achieve clear margins and followed closely for recurrence. Often, resection of the entire radiation field is required which can result in a large defect, and margins are not always clearly defined.^8^ for example, a case report by Yoon et al described a patient treated with radiation therapy for a peripheral nerve-sheath tumor, who was found to have AVLs outside the field of radiation exposure.^9^ Finally, there are few reported cases of good outcomes following Mohs surgery for angiosarcoma. A review by Rodriguez-Jimenez et al. states that over the last century only 1 patient has remained relapse free following Mohs for angiosarcoma, while all other patients had relapsed.^10^ Therefore, Mohs surgery can be considered as potential treatment modality to ensure clear margins in areas of concern, though should be utilized judiciously as this would not be appropriate for all cases of AVLs.

Herein, we describe a case of a patient with a history of breast cancer that underwent mastectomy and radiation therapy, and developed AVLs in her left reconstructed breast and subsequently underwent Mohs surgery for clearance of her lesions. Mohs surgery can be considered as a treatment modality for AVLs given the potential for significant subclinical spread. Mohs surgeons should be aware of this pathology and the risk of malignant potential to angiosarcoma.

## Conflicts of interest

None disclosed.
